# Deaths among children with life-limiting or life-threatening conditions in Wales: a full population cohort

**DOI:** 10.1136/archdischild-2025-329153

**Published:** 2025-10-30

**Authors:** Andre Bedendo, Stuart Jarvis, Richard D W Hain, Lorna K Fraser

**Affiliations:** 1 Department of Health Sciences, University of York, York, UK; 2 Noah's Ark Children's Hospital for Wales, Cardiff, UK; 3 Cicely Saunders Institute, King’s College London, London, UK

**Keywords:** Mortality, Epidemiology, Palliative Care

## Abstract

**Objectives:**

To describe trends in demographic and clinical characteristics of children with a life-limiting condition (LLC) who died in Wales.

**Methods:**

Linked full population cohort observational study of all children and young people (CYP) aged 0–25 years old with an LLC, resident in Wales from 2003 to 2020. Deaths were identified using four data sources: death registries, congenital anomaly, inpatient and day case activity, and accident/emergency attendances.

**Results:**

Of the 6828 recorded deaths, 39% (N=2633) had an LLC-related diagnosis. The proportion of LLC deaths increased from 36% to 42% from 2003 to 2008 to 2015to 2020, with 43% (1135/2633) of LLC-related deaths occurring among children aged up to a year old. Among 5–11 years old, 68% (276/406) had an LLC diagnosis on their death certificate. Of all deaths with an LLC-related diagnosis, 28% (738/2633) occurred in the most deprived quintile areas. Neurological conditions were the most common LLC diagnostic group (22%, 570/2633). Among those with an LLC, 63% (1654/2633) had the condition reported as cause of death (*Underlying*: 39% (1017/2633); *Related*: 24% (637/2633)).

**Conclusions:**

The proportion of LLC-related deaths in Wales increased 6% from 2003 to 2020. LLC-related deaths accounted for nearly 40% of total deaths in CYP up to 25 years. One-third of these deaths did not mention a life-limiting diagnosis on the death certificate. Seventy per cent of deaths among the 5–11 age group had a life-limiting diagnosis present.

**WHAT IS ALREADY KNOWN ON THIS TOPIC**

- From 2002 to 2007, 54% of childhood deaths (ages 0–19) in Wales were linked to life-limiting conditions (LLCs).

**WHAT THIS STUDY ADDS**

- Although the number of children (0–25 years) deaths in Wales from 2003 to 2020, the proportion of LLC deaths increased by 6%.
- LLC-related deaths accounted for nearly 50% of deaths up to age 18 and 40% up to age 25.

**HOW THIS STUDY MIGHT AFFECT RESEARCH, PRACTICE OR POLICY**

- The new estimates can help shape service planning and delivery.
- Our findings reinforce the importance of sustainable funding for palliative care services to support children with LLC and their families, who now account for a larger share of child mortality.

## Introduction

 Although child mortality has decreased over the last few decades, around 3100 infants and children die in England and Wales every year.^1^ Approximately half of the deaths are reported as occurring from underlying life-limiting conditions,^2^ with congenital malformations and chromosomal abnormalities reported as the leading cause of death from ages 1–4 years in England and Wales.^3^ Life-limiting conditions (where there is no reasonable hope for cure and from which children will die, such as Duchenne muscular dystrophy) and life-threatening conditions (where a curative treatment may be feasible but can fail, such as cancer), hereafter referred to as LLC.^4^

The most recent estimates for the UK reports to 86 625 children (0–19 years) living with an LLC in England (2017/2018) and 15 404 in Scotland (2013/2014).^5 6^ These studies also showed that estimates have increased markedly over the last decade. We recently estimated a total of 3655 children aged up to 18 years (0–18 years) and 4289 children up to 25 years (0–25 years) living in Wales in 2019 had an LLC.^7^ When compared to 2009, these figures represent a 22.8% increase for up to 18 years and 29.8% increase in up to 25 years.

Considering the large affected population and the high proportion of child deaths that are related to LLC, service planning and delivery necessitate the most up-to-date figures. It has been previously estimated that 54% of all childhood deaths (0–19 years) from 2002 to 2007 in Wales were caused by LLC.^8^

This study aimed to describe demographic and clinical characteristics of children with an LLC who died in Wales from 2003 to 2020 using a full population cohort.

## Methods

### Design

Observational study of a whole population cohort.

### Participants

All children and young people aged 0–25 years old with an LLC, resident in Wales from 2003 to 2020.

### Data sources and outcomes

The main source of data is the SAIL databank,^9^ which provides access to linked health and administrative data for Wales (www.saildatabank.com).

1. Deaths were recorded in several datasets.
2. Annual District Death Extract (ADDE)—Register of all deaths relating to Welsh residents, including those that died out of Wales.
3. Congenital Anomaly Register and Information Service (CARIS)—Collects information about any fetus or baby who has or is suspected of having a congenital anomaly and whose mother is normally resident in Wales at time of birth. An anomaly is defined as involving structural, metabolic, endocrine or genetic defects, present in the child/fetus at the end of pregnancy, even if not detected until after the birth. It includes babies in whom anomalies are diagnosed at any time from conception to the end of the first year of life. Conditions in CARIS are not limited to LLC.
4. Patient Episode Database for Wales (PEDW)—The database contains all inpatient and day case activity undertaken in NHS Wales plus data on Welsh residents treated in English Trusts. The basis of a patient’s consultant episode, which is the time an admitted patient spends in the continuous care of one consultant within one NHS provider.
5. Emergency Department Dataset (EDDS)—Attendance and clinical information for all Accident and Emergency attendances.

Children with an LLC were identified using International Classification of Diseases 10th Revision (ICD10) diagnoses and Read codes for LLC.^10 11^ The list was built on previously existing lists of LLC diagnoses^5 11^ and was further refined and expanded by translating the ICD10 coding framework into Read Codes. LLC were categorised as neurologic, haematologic, oncologic, metabolic, respiratory, circulatory, gastrointestinal, genitourinary, perinatal, congenital, other (eg, ophthalmologic, rheumatologic/autoimmune, musculoskeletal/bone disorders, transplant medicine and general medical/palliative care). Full details and code list can be found elsewhere.^7^ Diagnoses and codes were searched in the following ways:

1. Cause of death underlying or related presented in the death certificates. Death certificates are completed by a qualified medical practitioner^12^ and list the underlying cause of death (1) the disease or injury which initiated the train of morbid events leading directly to death, or (2) the circumstances of the accident or violence which produced the fatal injury. It also lists any other disease, injuries, conditions or events that contributed to the death. Any information listed as secondary or as any other cause of death was considered a related cause of death.
2. Hospital admissions: inpatient and day cases.
3. Accident and emergency attendances.
4. Congenital anomaly register data.

### Data analysis

We used frequencies and proportions to describe the variables. To comply with the reporting requirements from the SAIL databank, low counts (10 or less) were masked when appropriate.

Participants were categorised under different age groups: 0–17 and 0–25 years. For more detailed analyses, multiple age bands were used: 0–27 days, 28 days–1 year, 1–4 years, 5–11 years, 12–17 years, 18–25 years.

Lower Super Output Area (2011) values were determined and mapped to Welsh Index of Multiple Deprivation 2019 scores using data from The Welsh Government, and then split into five equal population (0–25 years population) groups.^7^

## Results

We identified a total of 6828 recorded deaths, of which 2633 (39%, 2633/6828) had an LLC-related diagnosis (reported in admissions or in cause of death; online supplemental figure S1). This proportion is higher among those 0–18 years old (48%, 1950/4088).

**Figure 1:**
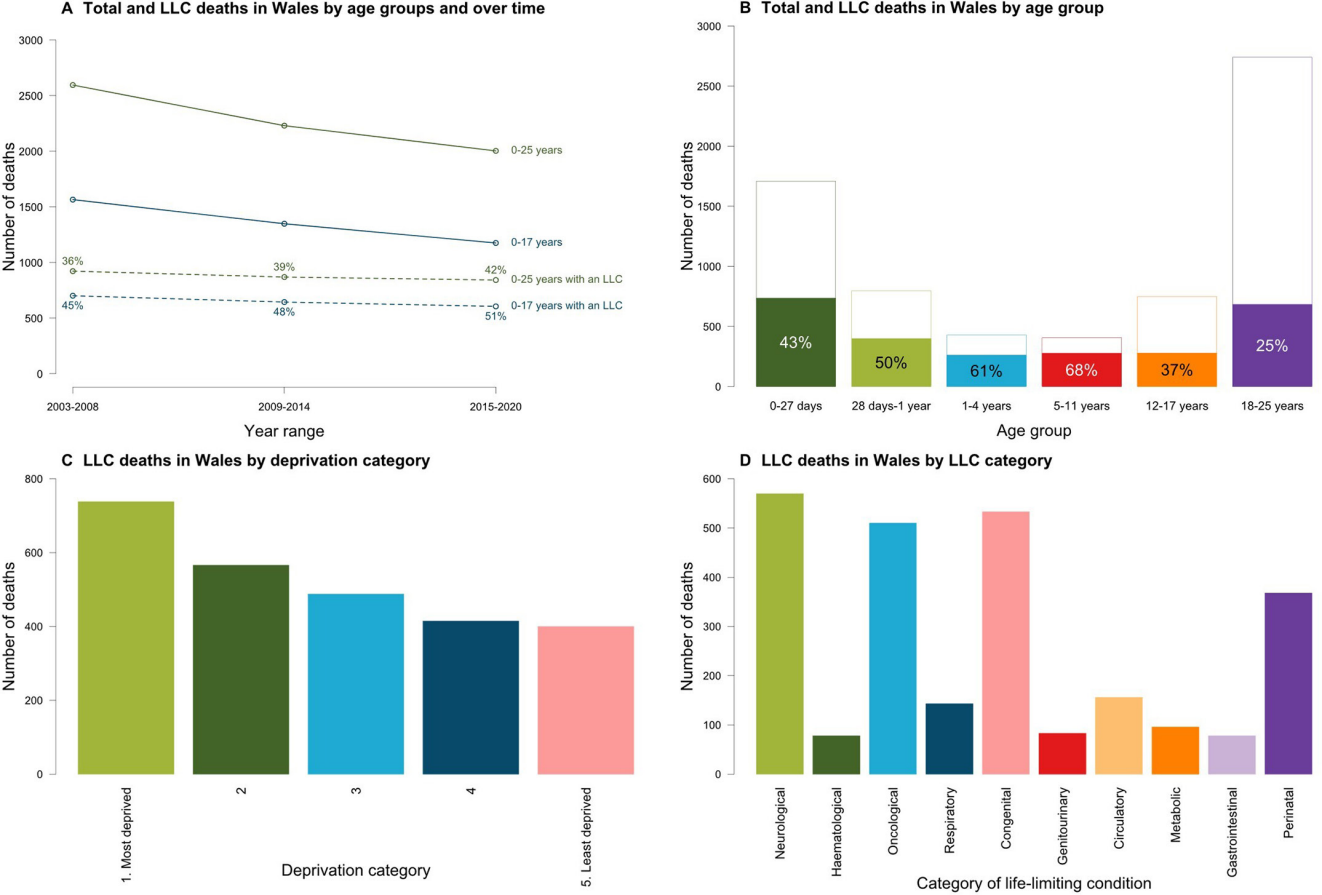
(Panel A) Deaths in Wales from 2003 to 2020 for children (0–18 years) and children and young people (0–25 years) with and without evidence of a life-limiting condition (LLC) being present. Numbers adjacent to points indicate the proportion of deaths in the year that were for people with an LLC. (Panel B) Deaths in Wales by age group with (shaded parts of bars) and without (unshaded parts of bars) an LLC being recorded as present for 2003–2020. Overall bar height gives the total deaths in that age group for 2003–2020. Numbers on bars are the proportion of deaths in each age group that were for people with LLCs. (Panel C) Deaths in Wales for children and young people (0–25 years) by deprivation category. Bar height gives total deaths in the category for 2003–2020. (Panel D) Categories of LLC recorded for children and young people in Wales known to have died from 2003 to 2020 (categories are non-exclusive—a death may be recorded under multiple categories).

Figure 1A shows that despite the overall number of deaths decreasing over time from 2003 to 2008 to 2015–2020, the proportion of LLC deaths increased during the same period (from 36% (922/2595) to 42% (842/2003)).

### Demographic characteristics

Figure 1B presents deaths by age group and the respective proportion of deaths in each age group that were for people with LLCs. Despite most of the total deaths occurring among children aged 18–25 years (40%, 2740/6828), only a quarter of LLC deaths in this age group was the lowest (25%). The proportions of LLC deaths over the total number of deaths were at the highest among 28 days–1 year (50%, 399/797), 1–4 years (61%, 261/429) and 5–11 years (68%, 276/406). Neonates (0–27 days) accounted for 25% (1708/6828) of all deaths, but LLC deaths account for 43% of all deaths in this age group (figure 1B). The proportion of deaths was less frequent among older children (12–17 years: 37% (278/748)) and young adults (18–25 years: 25% (683/2740)).

Online supplemental table 1 presents detailed proportions of LLC and non-LLC deaths by year of death and age group. When only LLC deaths are considered, neonates accounted for the largest proportion of deaths (28%, 736/2633), followed by children aged 28 days–1 year (15%, 399/2633). The proportions were around 10% for the age groups 1–4 years (261/2633), 5–11 years (276/2633) and 12–17 years (278/2633) and slightly higher (~13%) among those aged between 18–21 years (332/2633) and 22–25 years (351/2633).

The number of LLC-related diagnosis deaths was generally stable over time among neonates and non-neonates. However, compared with the initial period, there was a reduction in the percentage of deaths among those aged 28 days–1 year (17% to 12%) and an increase among children aged 22–25 years (11% to 16%).

More deaths occurred in the most deprived areas (28%, 738/2633), and the percentage gradually decreased down to 15% (400/2633) among least deprived areas (figure 1C).

### Clinical characteristics

Neurological conditions were the most common diagnosis (22%, 570/2633), followed by congenital (20%, 533/2633) and oncological (19%, 510/2633) (figure 1D). The least common diagnoses were genitourinary (3%, 83/2633), haematological (3%, 78/2633) and gastrointestinal (3%, 78/2633).

Detailed information by year of death (ranges) is presented in online supplemental table 2. Over the years, there was an increase in the proportions for neurological, haematological and genitourinary conditions. On the contrary, a decrease was observed for oncological, respiratory and circulatory conditions. Over the years, the proportions remained largely flat for congenital, metabolic, gastrointestinal and perinatal conditions.

### Causes of death among those with LLC

Among those identified with an LLC, 63% (1654/2633) had the condition reported as the cause of death (*Underlying cause of death:* 39% (1017/2633) or *Related cause of death:* 24% (637/2633)) in death certificates. LLC was not mentioned in 33% (879/2633) of death certificates, and 4% (100/2633) were trauma-related deaths (online supplemental table 2).

Among neonates, LLC was reported as the underlying cause of death in only 5% (38/736) of the causes of deaths, and mentioned as a related cause of death in 56% (411/736) of the registries. About 54% of deaths among 28 days–1 year and 1–4 years age groups had LLC reported as the underlying or related cause of death. These figures increased with age, and the LLC was more frequently mentioned as the underlying cause of death among those aged 22–25 years (60%, 210/351). Reports of trauma-related deaths also increased with age and reached the highest proportions among those aged 22–25 years, corresponding to 9% (30/351) of all deaths in this age group.

Regarding the deprivation category, proportions of reports of LLC as cause of death were largely similar across the groups. Despite no particular trends being observed, reports of LLC as an underlying cause of death were slightly less reported among the most deprived category (35%, 258/739), compared with other categories (ranging from 39% to 42%).

When considering the diagnostic category, 85% (431/510) of all oncological conditions were reported as underlying causes of death. This was followed by 59% (57/96) of all metabolic and 50% (39/78) of the haematological conditions. Perinatal (9%, 34/368) and genitourinary (13%, 11/83) conditions were the least commonly reported as the underlying cause of death.

## Discussion

Between 2003 and 2020 in Wales, around 48% of deaths among children 0–18 years old and 39% among those 0–25 years were associated with LLC diagnoses. Although the total number of deaths declined over this period, the proportion of LLC deaths increased by 6%. Nearly 70% of deaths among those aged between 5 and 11 years old had an LLC-related diagnosis. Neurological conditions were the most common LLC-related diagnosis, followed by congenital and oncological conditions. The LLC condition was not mentioned in a third of reported deaths.

This varied by age with 43.1% of neonates who died having an LLC and peaking at 68% in 5–11 years old. LLC deaths accounted for a higher percentage of deaths over time (36% in 2003–2008 to 42% in 2015–2020) despite a 23% decrease in the total number of deaths. On death certificates, LLC was recorded as the underlying or related cause of death in 63% of our sample and using death certificate data only to identify the LLC condition would have missed a third of the LLC cases.

Compared with a previous study, specific analysis using a subsample of children 0–19 years showed a higher number of deaths from 2003 to 2007 (N=1532) in our study compared with 1052 for the same ages and from 2002 to 2007.^8^ Our study also showed a lower percentage of deaths with an LLC-related diagnosis. The differences may relate to an underreporting of neonatal causes of death.

Based on death certificate data, LLC was less commonly reported as the underlying cause of death among neonates. This is likely the neonates’ cause of death being coded differently and the limited availability of this information on the datasets. The LLC was not mentioned as a cause of death of around 40% of children from 28 days–4 years. This reduces to about 20% among those aged 22–25 years. As expected, trauma-related causes of death were more commonly reported among older age bands.^13^ Our results support that death certificates do not adequately reflect LLC estimates and highlight the relevance of initiatives to improve the quality and accuracy of death certificates in the UK.

Similar to our findings using English and Scottish data,^13^ our results confirmed oncological and haematological diagnoses as the most common LLCs reported as underlying cause of death on death certificates, and genitourinary diagnoses as most commonly not mentioned in death certificates.

As expected, there were more LLC-related deaths in most deprived areas and fewer in least deprived areas. This has also been reported previously in Wales^14^ as well as in England and Scotland.^13^

### Strengths and limitations

This study used multiple national data collected using established formal processes to identify deaths related to LLC diagnoses to ensure representativeness of the children population of Wales. We also used a systematic approach to derive our main results, which have been previously tested. This improves comparability to other studies.

The main limitation of this study is a possible underestimation of the neonates’ causes of death reporting an LLC. This is a consequence of how data are recorded, data availability and limited ability of the ICD10 and Read codes framework to fully capture the conditions among this age group.

## Conclusions

In Wales from 2003 to 2020, nearly half of the deaths among children 0–18 years old and 4 in every 10 among those 0–25 years old had a related life-limiting diagnosis. Although there was a decline in the total number of deaths during the same period, the proportion of LLC deaths increased by 6%. These findings reinforce the importance of sustainable funding for palliative care services that support children with LLC and their families. Nearly 70% of deaths among those aged between 5 and 11 years old had an LLC-related diagnosis. Neurological conditions were the most common LLC-related diagnosis reported. One-third of these deaths did not mention a life-limiting diagnosis on the death certificate.

## Supplementary material

10.1136/archdischild-2025-329153 online supplemental file 1

## Data Availability

Data may be obtained from a third party and are not publicly available.
