# Supplementary material for: Deaths among children with life-limiting or life-threatening conditions in Wales: a full population cohort
Source: Arch Dis Child. 2025 Oct 30;111(3):e329153. doi: 10.1136/archdischild-2025-329153 (PMC13018728; doi:10.1136/archdischild-2025-329153)
Supplement: online supplemental file 1 [file archdischild-111-3-s001.docx]

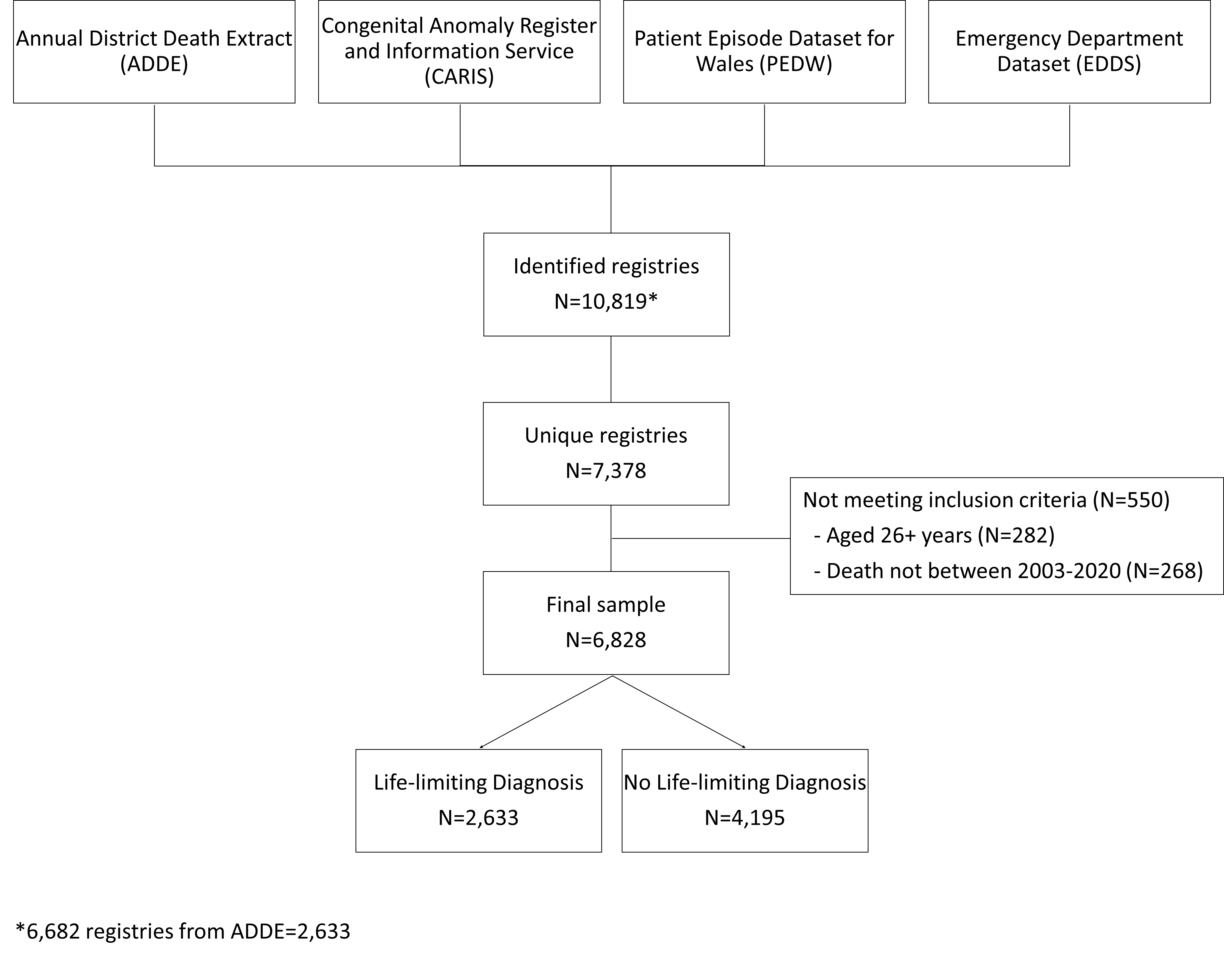


**Supplementary Figure S1 – Sample identification flow diagram.**

**Supplementary Table 1** **Life-limiting condition (LLC) and non-LLC deaths by year of death (ranges) and age group.**

|  | ***2003-2008*** | | | ***2009-2014*** | | | ***2015-2020*** | | | **Overall** | | |
| --- | --- | --- | --- | --- | --- | --- | --- | --- | --- | --- | --- | --- |
|  | **Total (N=2595)** | **No LLC (N=1673)** | **LLC (N=922)** | **Total (N=2230)** | **No LLC (N=1361)** | **LLC (N=869)** | **Total (N=2003)** | **No LLC (N=1161)** | **LLC (N=842)** | **Total (N=6828)** | **No LLC (N=4195)** | **LLC (N=2633)** |
| Age Group |  |  |  |  |  |  |  |  |  |  |  |  |
| 0-27 days | 620 | 370 (60%) | 250 (40%) | 579 | 321 (55%) | 258 (45%) | 509 | 281 (55%) | 228 (45%) | 1708 | 972 (57%) | 736 (43%) |
| 28 days - 1 year | 310 | 155 (50%) | 155 (50%) | 278 | 134 (48%) | 144 (52%) | 209 | 109 (52%) | 100 (48%) | 797 | 398 (50%) | 399 (50%) |
| 1-4 years | 166 | 69 (42%) | 97 (58%) | 128 | 56 (44%) | 72 (56%) | 135 | 43 (32%) | 92 (68%) | 429 | 168 (39%) | 261 (61%) |
| 5-11 years | 149 | 49 (33%) | 100 (67%) | 131 | 48 (37%) | 83 (63%) | 126 | 33 (26%) | 93 (74%) | 406 | 130 (32%) | 276 (68%) |
| 12-17 years | 320 | 221 (69%) | 99 (31%) | 232 | 145 (62%) | 87 (38%) | 196 | 104 (53%) | 92 (47%) | 748 | 470 (63%) | 278 (37%) |
| 18-25 years | 1030 | 809 (79%) | 221 (21%) | 882 | 657 (74%) | 225 (26%) | 828 | 591 (71%) | 237 (29%) | 2740 | 2057 (75%) | 683 (25%) |

**Supplementary Table 2 Life-limiting condition deaths** **by year of death (ranges), demographic, cause of death and diagnostic category**

|  | 2003-2008 | 2009-2014 | 2015-2020 | Total |
| --- | --- | --- | --- | --- |
| **Deaths in period** |  |  |  |  |
| - Total deaths | 922 (100.0%) | 869 (100.0%) | 842 (100.0%) | 2633(100%) |
| **Age Group** |  |  |  |  |
| - 0-27 days | 250 (27%) | 258 (30%) | 228 (27%) | 736(28%) |
| - 28 days - <1 year | 155 (17%) | 144 (17%) | 100 (12%) | 399(15%) |
| - 1-4 years | 97 (11%) | 72 (8%) | 92 (11%) | 261(10%) |
| - 5-11 years | 100 (11%) | 83 (10%) | 93 (11%) | 276(11%) |
| - 12-17 years | 99 (11%) | 87 (10%) | 92 (11%) | 278(11%) |
| - 18-21 years | 120 (13%) | 106 (12%) | 106 (13%) | 332(13%) |
| - 22-25 years | 101 (11%) | 119 (14%) | 131 (16%) | 351(13%) |
| **Deprivation category** |  |  |  |  |
| - 1. Most deprived | 272 (30%) | 232 (27%) | 234 (28%) | 738(28%) |
| -2 | 196 (22%) | 186 (22%) | 184 (22%) | 566(22%) |
| -3 | 172 (19%) | 153 (17%) | 163 (20%) | 488(19%) |
| -4 | 131 (14%) | 144 (17%) | 140 (17%) | 415(16%) |
| - 5. Least deprived | 137 (15%) | 147 (17%) | 116 (14%) | 400(15%) |
|  |  |  |  |  |
| **Cause of death categories** |  |  |  |  |
| - LLC Underlying CoD | 398 (43%) | 291 (34%) | 328 (39%) | 1017(39%) |
| - LLC Related CoD | 221 (24%) | 224 (26%) | 192 (23%) | 637(24%) |
| - Trauma Related CoD | 25 (3%) | 31 (4%) | 44 (5%) | 100(4%) |
| - LLC not mentioned in CoD | 278 (30%) | 323 (37%) | 278 (33%) | 879(33%) |
| **Diagnostic category** |  |  |  |  |
| - Neurological | 193 (21%) | 178 (21%) | 199 (24%) | 570(22%) |
| - Haematological | 19 (2%) | 23 (3%) | 36 (4%) | 78(3%) |
| - Oncological | 191 (21%) | 163 (19%) | 156 (19%) | 510(20%) |
| - Respiratory | 59 (6%) | 48 (6%) | 36 (4%) | 143(6%) |
| - Congenital | 190 (21%) | 169 (20%) | 174 (21%) | 533(20%) |
| - Genitourinary | 13 (1%) | 30 (4%) | 40 (5%) | 83(3%) |
| - Circulatory | 69 (8%) | 60 (7%) | 27 (3%) | 156(6%) |
| - Metabolic | 34 (4%) | 32 (4%) | 30 (4%) | 96(4%) |
| - Gastrointestinal | 27 (3%) | 26 (3%) | 25 (3%) | 78(3%) |
| - Perinatal | 119 (13%) | 133 (15%) | 116 (14%) | 368(14%) |
| - Other | * | * | * |  |

LLC: Life-limiting condition; CoD: Certificate of Death
* Values masked due to low counts.
